# Use of Mobile and Wearable Artificial Intelligence in Child and Adolescent Psychiatry: Scoping Review

**DOI:** 10.2196/33560

**Published:** 2022-03-14

**Authors:** Victoria Welch, Tom Joshua Wy, Anna Ligezka, Leslie C Hassett, Paul E Croarkin, Arjun P Athreya, Magdalena Romanowicz

**Affiliations:** 1 Department of Molecular Pharmacology and Experimental Therapeutics Mayo Clinic Rochester, MN United States; 2 Department of Psychiatry and Psychology Mayo Clinic Rochester, MN United States; 3 Department of Clinical Genomics Mayo Clinic Rochester, MN United States; 4 Mayo Clinic Libraries Mayo Clinic Rochester, MN United States

**Keywords:** mobile computing, artificial intelligence, wearable technologies, child psychiatry

## Abstract

**Background:**

Mental health disorders are a leading cause of medical disabilities across an individual’s lifespan. This burden is particularly substantial in children and adolescents because of challenges in diagnosis and the lack of precision medicine approaches. However, the widespread adoption of wearable devices (eg, smart watches) that are conducive for artificial intelligence applications to remotely diagnose and manage psychiatric disorders in children and adolescents is promising.

**Objective:**

This study aims to conduct a scoping review to study, characterize, and identify areas of innovations with wearable devices that can augment current in-person physician assessments to individualize diagnosis and management of psychiatric disorders in child and adolescent psychiatry.

**Methods:**

This scoping review used information from the PRISMA (Preferred Reporting Items for Systematic Reviews and Meta-Analyses) guidelines. A comprehensive search of several databases from 2011 to June 25, 2021, limited to the English language and excluding animal studies, was conducted. The databases included Ovid MEDLINE and Epub ahead of print, in-process and other nonindexed citations, and daily; Ovid Embase; Ovid Cochrane Central Register of Controlled Trials; Ovid Cochrane Database of Systematic Reviews; Web of Science; and Scopus.

**Results:**

The initial search yielded 344 articles, from which 19 (5.5%) articles were left on the final source list for this scoping review. Articles were divided into three main groups as follows: studies with the main focus on autism spectrum disorder, attention-deficit/hyperactivity disorder, and internalizing disorders such as anxiety disorders. Most of the studies used either cardio-fitness chest straps with electrocardiogram sensors or wrist-worn biosensors, such as watches by Fitbit. Both allowed passive data collection of the physiological signals.

**Conclusions:**

Our scoping review found a large heterogeneity of methods and findings in artificial intelligence studies in child psychiatry. Overall, the largest gap identified in this scoping review is the lack of randomized controlled trials, as most studies available were pilot studies and feasibility trials.

## Introduction

### Background

The global burden of mental illnesses is daunting. In 2013, mental illnesses, such as major depression (2nd), anxiety disorders (7th), schizophrenia (11th), dysthymia (16th), and bipolar disorder (17th), were listed as some of the leading causes of years lived with disability [1]. Unipolar major depression and self-inflicted injuries were at number 2 and 14, respectively, as the top contributors to the global burden of disease in 2020 [2]. Despite significant advances in medical research, there is a distinct deficiency in the detection and treatment of psychiatric disorders, especially in children and adolescents [3]. The subsequent ramifications are evident. For instance, in 10% of completed suicides, the adolescent victims had no previously recorded psychiatric diagnoses [4]. With the increasing adoption of measurement-based practices in child and adolescent psychiatry, large volumes of data are being generated from clinical trials and routine practice. Such well-characterized data may prove to be fertile opportunities for innovations in data science and artificial intelligence (AI) to potentially address the shortcomings and subsequently improve the burden of disease in these populations.

Current approaches for the evaluation of psychiatric disorders predominantly rely on physician-patient history taking, collateral information, and patients’ self-reported questionnaires rather than objective laboratory tests or neuroimaging biomarkers. As a result, contemporary psychiatric assessments are inaccurate and ineffective in providing a reliable and individualized assessment of symptoms [3]. With growing evidence of large data-driven approaches (eg, AI) to individualize diagnoses and treatment management of psychiatric disorders in adults [5-9], there are opportunities for such a paradigm in child and adolescent psychiatry. AI approaches simulate humans’ ability to problem solve, plan, reason, and recognize patterns [3]. In these processes, computers *learn* the abilities by processing massive data sets through multilayered mathematical models (algorithms) and training methodologies (eg, cross-validation) improves the AI model’s predictive confidence [10].

Broadly, the field of AI subsumes the methodological paradigms of computing science. First, machine learning refers to a programing approach in computer science in which the behavior of a program is not fully determined by an established code but can adapt its behavior (ie, learn) based on the data gathered [11]. Simple neural networks have been used in medicine since the early 1990s to interpret electrocardiograms (ECGs) [12], individualized predictions of antidepressant response, and diagnosis of myocardial infarction [13]. Second, deep learning is a particular variant of machine learning that is often modeled using artificial neural networks, which typically consist of interconnected nodes representing artificial neurons [11]. Deep learning has been used to design drugs, predict gene mutation expression, analyze histological examples, and read radiographic images [14,15]. Third, natural language processing (NLP) involves training computers to understand text and spoken languages or words in the way humans communicate [15]. NLP is well adopted in medicine, where it is used to extract structured text (eg, diagnosis and treatment context) from unstructured text (eg, electronic health records). Finally, reinforcement learning is a field of computing where computers can be trained to make decisions based on past and current data and a given context to maximize long-term outcomes. For example, reinforcement learning has been used in tailoring treatments for epilepsy and sepsis [16]. These recent AI applications provide new possibilities for AI use in specialty medical practice, while projecting future utility in general medical practice [10].

### Objectives

AI has been innovating and reshaping medicine but progress in psychiatry and child and adolescent psychiatry, in particular, is slow. Most previous research in psychiatry has focused on either NLP or the integration of various biomarkers to classify certain disorders such as heart conditions, epilepsy, and various types of cancer [11]. In addition, limited sustained collaboration among engineers, data scientists, and mental health providers has affected the slower adoption of these techniques in psychiatric practice in comparison with psychiatry and other medical specialties [10,17]. A review by Shatte et al [18] discussed the literature focused on adult mental health issues and machine learning applications. Our unique review aims to summarize the available research in child and adolescent psychiatry literature investigating machine learning technology and AI applications. The second aim is to characterize future opportunities in AI research in child and adolescent psychiatry.

The growing adoption of wearable technologies (eg, smart watches) not only helps passively collect large volumes of data but also opens the doors for using the data to enable remote diagnostic capabilities in child and adolescent psychiatry. Remote diagnosis and management of psychiatric diseases in children is crucial, given the shortage of trained mental health professionals.

Although wearable devices might support diagnosis and management in the future, they will not be able to replace health care professionals and their clinical observations. In this context, we sought to review studies that gathered data of child and adolescent patients with psychiatric disorders through practical means and used passively gathered data for various prediction mechanisms.

## Methods

This scoping review used PRISMA (Preferred Reporting Items for Systematic Reviews and Meta-Analyses) information (Multimedia Appendix 1) [19] as a guide and was organized according to the steps outlined in this section.

### Step 1: Developing a Research Question

We identified what was the extent of literature on the use of wearable devices in the form of AI in child and adolescent psychiatry. We aimed to gain insight into the following research objectives: (1) understand how wearable devices are being used in child psychiatry by exploring the types of devices that are being used, investigating how these devices are being used in child psychiatry (aid diagnosis, evaluate treatment efficacy, make algorithms to predict behavioral outcomes, etc), and researching which physiological signals are being measured by these devices and (2) identify how the clinical knowledge of various pediatric psychiatric disorders has been expanded through the use of AI, specifically wearable devices.

### Step 2: Literature Search

A comprehensive search of several databases was performed on June 25, 2021. The search was restricted from the year 2011 through the date the search was conducted. The results were limited to English language articles. Animal studies were excluded from this study. The databases searched were Ovid MEDLINE (≥1946) and Epub ahead of print, in-process and other nonindexed citations and daily (equivalent to PubMed); Ovid Embase (≥1988); Ovid Cochrane Central Register of Controlled Trials (≥1991); Ovid Cochrane Database of Systematic Reviews (≥2005); Web of Science Core Collection via Clarivate Analytics (≥1975); and Scopus via Elsevier (≥1788).

The search strategy was designed and conducted by an experienced librarian (LH), with input from the study investigators (APA and MR). Controlled vocabulary supplemented with keywords was used to search for studies. The actual strategy, listing all the search terms used and how they were combined, is available in Multimedia Appendix 2.

### Step 3: Study Selection

The study selection process was divided into two phases: (1) title and abstract screening and (2) full-text article screening. For the first phase, 2 reviewers (VW and JW) screened the articles and either excluded them or included them on the source list. The resulting source list was reviewed by 2 other reviewers (APA and MR). A full-text article review was then performed by 2 reviewers (VW and JW) and the final source list was created.

### Step 4: Charting the Data

Most of the study data were extracted by a single researcher (VW). Another researcher (JW) helped complete 1 column and reviewed the table after it was completed. The following information was included in the table: year, sample population (size and demographics), psychiatric diagnosis, age range, wearable device used, and the measured physiological symptoms. The studies were divided based on the participants’ main diagnosis: autism spectrum disorder (ASD), attention-deficit/hyperactivity disorder (ADHD), and internalizing disorders (IDs).

### Ethics and Dissemination

This proposed scoping review did not require ethics or institutional review board approval, as data were collected through the review of published peer-reviewed literature and gray literature. The results will be submitted for publication in an open-access peer-reviewed journal and presented at relevant medical and engineering conferences.

## Results

### Study Selection

**Figure 1:**
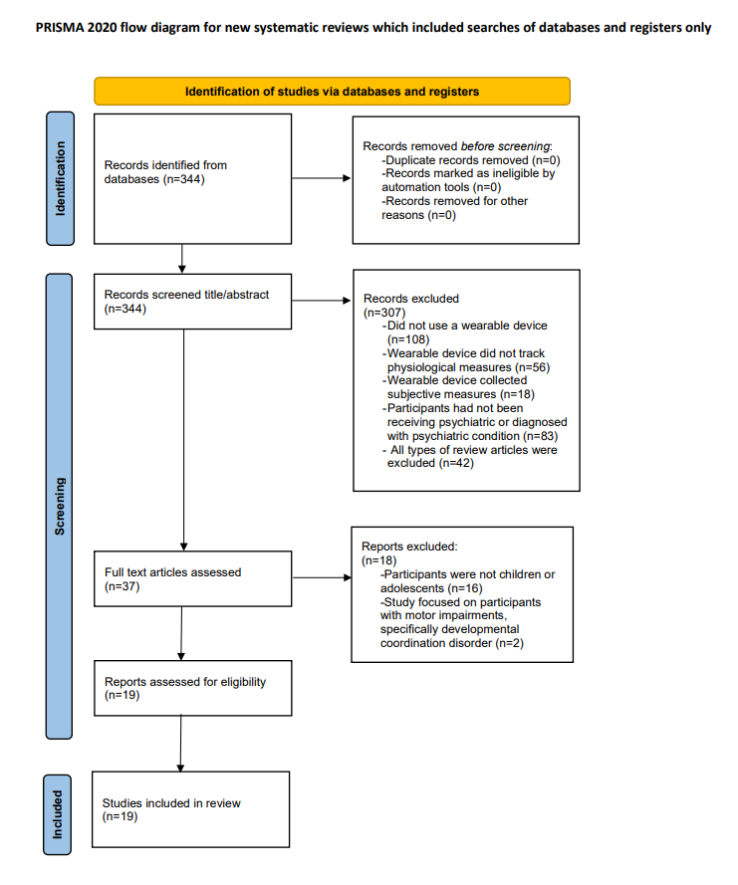
PRISMA (Preferred Reporting Items for Systematic Reviews and Meta-Analyses) flow diagram for study selection.

The initial search yielded 344 articles. In all, 2 researchers (VW and TJW) completed the title and abstract screening process to narrow down this list. They reviewed papers independently, and disagreements were reviewed by 2 additional researchers (APA and MR). During this step, 89.2% (307/344) articles were omitted because they did not satisfy the inclusion criteria. The criteria for inclusion of studies for review in the first round of screening were as follows: the study must use a wearable device that passively tracked physiological variables in real time (objective measurements) and the device must be worn by a patient who had been diagnosed with one or more of the following psychiatric disorders: oppositional defiant disorder, conduct disorder, mood disorder (depression, anxiety, and bipolar disorder), ADHD, learning disability (dyslexia, etc), autism, or psychotic disorder. A full-text screening of the remaining 10.8% (37/344) articles was conducted by the same 2 researchers (VW and TJW). Given our focus on child and adolescent psychiatric illnesses, the criteria for study inclusion were accordingly altered to focus on child and adolescent psychiatric patients only (aged 0-18 years), wherein studies were written in English and published before January 1, 2021. Eligible designs included randomized controlled trials, nonrandomized experimental studies, cohort studies, and case-control studies. In addition, studies that focused on motor impairment were excluded. The PRISMA flow diagram for the study selection process is illustrated in Figure 1. After the entire screening process was completed and reviewed by 2 additional researchers (APA and MR), 5.5% (19/344) articles were left on the final source list for this scoping review (Table 1).

**Table 1:** Summary of studies on the use of wearable devices in child psychiatry.

Study	Year	Sample characteristics	Diagnosis	Age	Device	Measured physiological symptoms
Bilecci et al [20]	2018	40 participants: 29 males and 11 females; race not specified	ASD^a^	18-36 months	ECG^b^ chest strap (Shimmer)	HR^c^, SDNN^d^, CV^e^, LF^f^, and HF^g^
Bilecci et al [21]	2016	5 participants: all males; race not specified	ASD	6-8 years	ECG chest strap and EEG^h^ headset (EEG- Enobio wireless device)	QEEG^i^ and HRV^j^
Di Palma et al [22]	2017	5 participants: all males; race not specified	ASD	6-8 years	ECG chest strap (based off Shimmer)	HR, RMSSD^k^, and RSA^l^
Faedda et al [23]	2016	155 participants: 97 males and 58 females; race not specified	BP^m^ or ADHD^n^	5-18 years	ActiGraph belt (AMI^o^ motionlogger)	Diurnal activity, sleep efficiency, and circadian regulation
Fioriello et al [24]	2020	24 participants: 18 males and 6 females; race not specified	ASD or LD^p^	30-72 months	ECG chest strap	HR
Gayet al [25]	2014	not specified	ASD	Not specified	Accelerometer wrist strap (Affectiva Q Sensor), EEG headset (MindWave Mobile), ECG chest strap (Zephyr BioHarness), and mobile phone app (MyMedia)	Skin temperature and skin conductive data (accelerometer), EEG power spectrums (EEG), and HR, HRV, respiration, body temperature, and respiration (ECG)
Goodwin et al [26]	2019	20 participants: 75% male; 95% White; 90% non-Hispanic	ASD	6-17 years	Wrist-worn biosensor (Empatica E4)	HRV, EDA^q^, and motion-based activity (accelerometer)
Krupa et al [27]	2016	60 participants: sex and race not specified	ASD	3-12 years	Wrist-worn biosensor	HRV and EDA or GSR^r^
Kushki et al [28]	2015	24 participants: 17 males and 7 females; race not specified	ASD	Not specified	ECG chest strap (Shimmer)	RR^s^ intervals
Leikauf et al [29]	2021	32 participants: 17 males and 15 females; race not specified	ADHD	8-17 years	Smart watch app (StopWatch)	Movement data (actigraphy via accelerometer)
Lin et al [30]	2020	30 participants: 11 males and 4 females with age-matched controls; race not specified	ADHD	5-9 years	Smart watch (Asus ZenWatch 3)	Angular velocity (gyroscope) and acceleration in axial direction (accelerometer)
McGinnis et al [31]	2021	63 participants: 57% female; 75% White, non-Latinx; 11% Asian or Pacific Islander; 11% African American; 3% biracial	IDs^t^	4-8 years	IMU^u^ chest strap and headband (3-Space Sensor; YEI Technology)	Acceleration and angular velocity
McGinnis et al [32]	2019	63 participants: 57% female; 65% White; 82.5% in 2-parent households; 32% income >US $100,000	IDs	3-8 years	IMU chest strap (3-Space Sensor; YEI Technology)	Acceleration and angular velocity
Min et al [33]	2011	4 participants: sex and race not specified	ASD	Not specified	Accelerometers worn on wrists, ankles, and upper body	Motion data (flapping, rocking, punching, and hitting)
Munoz-Organero et al [34]	2019	36 participants: 15 males and 3 females with nonmatching controls; race not specified	ADHD	6-16 years	Accelerometers worn on wrists and ankles (Runscribe inertial sensors)	Acceleration and movement patterns
Ouyang et al [35]	2020	10 participants: sex and race not specified	ADHD	5-11 years	Accelerometer embedded in a smart watch	Linear motion
Pfeiffer et al [36]	2019	6 participants: sex not specified; 4 White; 2 Latin American or Hispanic	ASD	8-16 years	Wrist-worn biosensor (Empatica E4)	Skin conductance levels and NS-SCRs^v^ (EDA data)
Redd et al [37]	2020	5 participants: sex and race not specified	IDs	8-12 years	Wrist-worn biosensor (Empatica E4)	HR, HRV, electrical property fluctuations in the skin (EDA data), motion (accelerometer), and peripheral skin temperature (infrared thermophile)
Wilson et al [38]	2021	5 participants: sex and race not specified	ASD	3-12 months	Ankle-worn biosensors (APDM Opal; APDM Wearable Technologies)	Motion complexity (accelerometer, gyroscope, and magnetometer)

^a^ASD: autism spectrum disorder.

^b^ECG: electrocardiogram.

^c^HR: heart rate.

^d^SDNN: SD of the averaged normal sinus RR intervals for 5-minute segments.

^e^CV: time interval between 2 consecutive R waves.

^f^LF: low frequency.

^g^HF: high frequency.

^h^EEG: electroencephalography.

^i^QEEG: quantitative electroencephalography.

^j^HRV: heart rate variability.

^k^RMSSD: root-mean square of the successive normal sinus RR interval difference.

^l^RSA: respiratory sinus arrhythmia (indicator of autonomic function).

^m^BP: blood pressure.

^n^ADHD: attention-deficit/hyperactivity disorder.

^o^AMI: acute myocardial infarction (motionlogger ActiGraph belt).

^p^LD: learning disability.

^q^EDA: electrodermal activity.

^r^GSR: galvanic skin response.

^s^RR interval, the time elapsed between 2 successive R waves of the QRS signal on the electrocardiogram.

^t^ID: internalizing disorder.

^u^IMU: inertial measurement unit.

^v^NS-SCR: nonspecific skin conductance response.

### Introductory Information

The studies on the final source list were published between 2011 and 2021, and were spread across the world with most being conducted in the United States. In addition, the growth rate of research in this field increased substantially after 2018. The following sections provide a synopsis of each of the sources organized into the 3 categories previously outlined.

### Studies Focused on ASD

Most studies (11/19, 58%) [20-22,24-28,33,36,38] focused on how the use of wearable devices could be used to aid in the treatment, behavioral prediction, and diagnosis of children with ASD. Although each study analyzed patients with ASD, the objectives and methods varied greatly among the studies. Several studies used ECG chest straps [20-22,24,25,28] for the most part to categorize the autonomic nervous system responses in patients with ASD during various tasks. For example, a study by Bilecci et al [20] used ECG strap during a joint attention stimuli in toddlers with ASD. The ECG chest strap measured SD of the average normal sinus RR intervals (the time elapsed between 2 successive R waves of the QRS signal on the ECG) for 5-minute segments, heart rate (HR), CV (time interval between 2 consecutive R waves), low frequency (changes in sympathetic regulation), and high frequency (changes in parasympathetic regulation). The results showed that the SD of the average normal sinus RR intervals for 5-minute segments, CV, and low frequency values were significantly higher in the ASD group than in the control group at baseline. In addition, the CV was significantly higher in the ASD group during the joint attention task. These findings suggest that joint attention tasks coupled with wearable devices could potentially help physicians diagnose autism in toddlers.

A longitudinal study by Di Palma et al [22] mediated sociocognitive tasks through serious games allowed for the coding of child behavior. Children received treatment for 6 months while being monitored by an ECG wearable chest belt. The serious games consisted of joint attention tasks and imitation exercises and the ECG belt measured HR, respiratory sinus arrhythmia (RSA; indicator of autonomic function), and root-mean square of the successive normal sinus RR interval difference. There was an increase in HR events during sociocognitive tasks. Researchers also found a correlation between detected physiological events and the level of involvement of the child during the task, along with a decrease in RSA and root-mean square of the successive normal sinus RR interval difference during activity which indicates proficient social interaction. Over time, patients displayed an increased percentage of physiological events associated with lower RSA during activity, which suggests improvement in cognitive engagement throughout the course of treatment. These predictive algorithms could be used at home by parents, at school by teachers, and in the clinic by therapists to create more individualized therapy plans.

Several studies used electroencephalography (EEG) in addition to ECG measures [21,25]. Bilecci et al [21] focused on obtaining quantitative EEG (pattern analysis of EEG) that was meant to determine treatment efficacy. Participants with ASD were monitored during a socioemotive interaction to implement a more individualized and effective treatment plan for these children. The results showed that all children yielded different measurements which emphasizes the importance of individualized therapy. The use of an AI device such as this would allow therapists to track a child’s engagement so that they can tailor their therapy to the child’s specific needs. Another study, in addition to measurement of EEG and ECG, also used an accelerometer wrist strap and created a mobile phone app called MyMedia and MySchedule [25]. The 6 main emotions that the app was designed to capture were happiness, sadness, fear, disgust, surprise, and anger. The sensors and facial recognition detected pupil dilation, skin conductance, HR or HR variability, blood pressure, concentration, and attention levels through a headset, watch, and chest strap. This method could provide a personalized way for autistic children and their caregivers to understand and manage their emotions.

Many of the studies used wrist-worn biosensors [26,27,36]. Some of the studies used accelerometers worn on various body parts such as wrists [25], ankles [38] as well as wrists, ankles, and upper body [33]. Using the Empatica E4 (Empatica, Inc) device study by Goodwin et al [26] investigated whether collecting and analyzing physiological and motion data from children with ASD during naturalistic observations could predict aggression. This wrist-worn sensor measured cardiovascular and electrodermal activity, along with detecting motion using an accelerometer. The results suggested that aggression to others can be predicted 1 minute before it occurs if biosensor data are collected for 3 minutes before the aggressive behavior. In this study, aggression was defined as hitting, kicking, biting, scratching, grabbing, pulling, pinching, or throwing objects at others. To make binary aggression predictions, a ridge-regularized logistic regression was used with the extracted time series features as input variables. This method had 84% average prediction accuracy and the average duration of aggressive episodes was 28 seconds.

Wilson et al [38] used wearable ankle sensors to diagnose ASD in children. Many believe that motor dysfunction may be predictive of ASD and the study used wearable ankle sensors to track full-day motor activity in infants with a high familial risk for ASD. These sensors contained a 3D-accelerometer, a 3D-gyroscope, and a 3D-magnetometer. Leg movement data were collected when the participants were aged 3, 6, 9, and 12 months, and an autism diagnostic tool was used to evaluate each child at the ages of 18 and 36 months. On the basis of the movement data collected from the sensors, the researchers were able to construct a new measure of motion complexity defined in terms of the variability of the frequency components underlying the observed movements. The results of the study showed that high-risk infants with a later diagnosis of ASD showed lower motion complexity compared those who were not diagnosed later. In fact, there was a stronger correlation between motion complexity and ASD outcome relative to cognitive ability and adaptive skills, making this method a promising diagnostic tool.

A study by Krupa et al [27] also used a wearable wristband that measured the galvanic skin response and HR variability (indicators of the autonomic nervous system) for diagnostic purposes. The machine was also used to determine a child’s current emotion. The results showed that the machine could differentiate children with ASD from normally developing children with 65% accuracy. In addition, it could differentiate neutral from emotion with 93.33% accuracy and happiness from involvement with 90% accuracy.

An intervention study by Pfeiffer et al [36] evaluated how well in-ear and overear headphones can decrease sympathetic activation in children with ASD and associated auditory hypersensitivity (hyperacusis) by measuring skin conductivity through electrodermal activity. Empatica E4 wristbands collected electrodermal activity data, as skin conductance is an indicator of stress or anxiety levels, and hyperacusis is associated with stress and anxiety. The results showed that in-ear and overear noise attenuating headphones led to a significant difference in both skin conductance levels and frequency of nonspecific conductance responses in subsequent phases of the study compared with the baseline measurements completed at the beginning of the study.

### Studies Focused on ADHD

A small percentage of the studies (5/19, 26%) [23,29,30,34,35] examined how the use of wearable devices could be used to accurately diagnose ADHD and evaluate the treatment efficacy of various strategies used to treat children with ADHD. Although each of the studies in this category had different methods and objectives and collected different physiological measurements, all the devices used in these studies used an accelerometer [23,29,30,34,35].

In all, 11% (2/19) of studies used a smart watch application [29,30]. Leikauf et al [29] conducted a pilot study on the efficacy of StopWatch, a smart watch application designed to track movement and provide visual and haptic feedback regarding the movement data collected for patients with ADHD. As this application collected movement data via an accelerometer, this study focused specifically on the hyperactivity aspect of ADHD. In a similar study, researchers used data collected from the gyroscope and accelerometer placed in a smart watch to analyze the movements of children with ADHD [30]. They compared the ADHD cohort to an age- and sex-matched control group to determine whether these types of measurements could be used to diagnose children with ADHD. After collecting data for 2 hours for 3 consecutive days in a naturalistic setting, the data suggested that children with ADHD had more variable and frequent movements than the controls. The Zero-Crossing Rate values across all 3 axes of the gyroscope were higher in the ADHD group; however, only the variance across the y-axis yielded a significant difference.

Munoz-Organero et al [34] used 4 triaxial accelerometers (placed on both wrists and ankles) to analyze movement patterns in normally developing children; patients with ADHD, who were medicated; and patients with ADHD, who were nonmedicated. They then used the data collected to propose a recurrent neural network to characterize the movement patterns of normally developing children that can be used to assess the similarity of new patients, which could potentially direct diagnosis. The results demonstrated that patients with ADHD, who were nonmedicated, showed higher differences medium intensity movements compared with normally developing patients whereas patients with ADHD, who were medicated, showed different behavior in their low intensity movements.

The final study in this category aimed to develop an objective method of evaluating the therapeutic effects of ADHD treatments by placing an accelerometer in their smart watch that recorded the movements of these patients [35]. The variance values of the accelerometer data before and after 1 month of using the medication methylphenidate were compared to determine the treatment efficacy of this drug. The Swanson, Nolan, and Pelham questionnaire (a subjective measure of treatment efficacy) was then compared with the accelerometer results, and the correlation between the 2 measurements was moderately strong. In addition, the variance values along the y- and z-axes of the accelerometers significantly decreased after 1 month of medication use, suggesting that the medication helped patients with ADHD.

A study by Faeda et al [23] used an ActiGraph belt (acute myocardial infarction motionlogger) and aimed to determine whether measures of activity, sleep, and circadian rhythm could be used to differentiate pediatric patients with bipolar disorder from pediatric patients with ADHD. They compared three different study groups: children with ADHD, children with bipolar disorder, children with ADHD and a comorbid depressive disorder and typically developing children. Each of these groups of children wore an ActiGraph belt for 3 to 5 days, which measured arousal, circadian rhythms, and sleep wakefulness cycles. The results showed that sleep duration and circadian strength measurements differed between children with ADHD and those with bipolar disorder. In addition, children with bipolar disorder had reduced measures of total sleep, reduced relative circadian amplitude, and increased nocturnal activity relative to the control group as well as both ADHD groups. This study suggests that wearable devices and AI may aid in the diagnosis of these overlapping pediatric disorders.

### Studies Focused on IDs

A few studies (3/19, 16%) [31,32,37] investigated the identification children with IDs using wearable devices and predicted their adverse behavioral outcomes through machine learning and AI.

McGinnis et al [31] aimed to develop a digital phenotype for childhood internalizing psychopathology based on data collected from a wearable inertial sensor. Data were recorded while the child completed three different tasks: the bubbles task (induced positivity), snake task (induced anxiety), and speech task (induced fear). The sensors were placed on both the head and waist of the child and acceleration and body movements were measured with an inertial measurement unit. They found that the children with IDs *burned out* quicker during the bubbles task and that it helped identify depressive, anxious, and trauma-related disorders. On the other hand, the snake task helped identify children with withdrawn, anxious, and depressive problems, oppositional problems, and specific phobias. Similarly, the speech task identified children with withdrawn, anxious, and depressive problems. After analyzing these results, they found that the phenotype from features that measured reward responsiveness could accurately detect children with underlying internalizing psychopathology with 75% accuracy.

Redd et al [37] investigated whether tracking physiological signals, such as HR, skin electrodermal activity, and skin temperature, could help predict a meltdown to facilitate earlier and more effective intervention. These measurements were recorded using a wrist-worn biosensor that incorporated blood volume pulsivity measurements, an electrodermal activity sensor to measure electrical fluctuations in the skin, a 3-axis accelerometer to measure overall motion and activity, and an infrared thermophile used to measure peripheral skin temperature. Parents also recorded their observations, and then the observations and physiological data were compared to create a predictive algorithm. The results showed that this model can accurately classify the behavioral states of children with 68% accuracy; however, only 4 meltdowns were recorded during the study which means that more data need to be collected and analyzed.

## Discussion

### Principal Findings

The primary outcome of this scoping review was a characterization of 19 studies of child and adolescent patients with ASD, ADHD and ID that gathered data through practical means and used passively gathered data for various prediction mechanisms.

From the included studies we were able to provide a descriptive analysis of currently available devices used to passively gather data in AI trials. Most of the studies used either ECG strap or wrist-worn biosensor. This is somewhat surprising as, for example. studies by Fioriello et al [24] and Kushki et al [28] could only collect data on HR via ECG chest strap, whereas many of the commercially available wrist watches can collect significantly more data [39].

The information obtained from this review can guide future trial development. Only 1 trial [23] had a high number of participants (N=155). Other trials [33] managed to enroll only 4 participants. We were not able to find any trials on children and adolescents specifically with major depressive disorder or suicidality. Only 1 trial [23] compared children with ADHD and children with bipolar disorder; however, no studies specifically addressed children with bipolar disorder or psychosis. There were also no trials that gathered data during inpatient hospitalization or during evidence-based outpatient treatment that children would typically receive. No studies obtained data from parents as well during the trials focusing only on children.

All the studies summarized focused on detecting behavioral changes in patients. This leaves opportunities to innovate with the data to actuate the detected behaviors using reinforcement learning approaches. Particularly, if a state of behavior is predicted before it visibly manifests, there is an opportunity to alert responsible adults nearby to intervene using proven interventions. Such timely interventions may prove to be a positive reinforcement for children, who otherwise are penalized for unacceptable behavior. In older patients (ie, adolescents vs young children), physiology-based triggers (eg, based on HR variability) with an action message (eg, *performing breathing exercises for 30 seconds*) may be helpful in forcing a positive change in behavior or mood.

Feature extraction and feature engineering are the key aspects of machine learning and AI efforts using wearable data [40] Given the heterogeneity in the types of devices and clinical questions being researched, approaches to extract signals from wearable data widely differed among the literature reviewed in this work. Feature extraction methods (eg, principal-component analysis, locally linear embedding, and autoencoding) will facilitate the identification of wearable-derived measures associated with clinical diagnosis or outcomes. Feature engineering uses wearable-derived measures to develop features for downstream analyses using domain-guided knowledge (eg, deriving motion features from raw accelerometer data). Determining the feature extraction or engineering approach is dependent on the nature of the clinical question being posed and types of measurements derived from wearables.

As AI in child and adolescent psychiatry is an emerging field, there are obvious gaps that will continue to be explored. Most of the papers focused on using the technology for diagnostic purposes, specifically ASD and ADHD. We believe that these emerging technologies can be used in other diagnostic categories such as mood, anxiety, and psychosis. There is also much room to innovate in the realm of intervention, treatment, and public health.

### Limitations

Our study had several limitations. We did not conduct a systematic review or prospectively register a protocol. This was expected to be a nascent area; hence, a scoping review was most appropriate. Another limitation is the potential that we have missed important original literature on the use of mobile and wearable AI in child psychiatry. This was mitigated by an extensive search of multiple databases, searching references of included articles, and ensuring duplicate review of all the abstracts and full-text. We chose not to include nonoriginal or non–peer-reviewed research and non-English articles. This might have led to us missing key conclusions drawn from this research. We also did not examine each of the machine learning technologies in detail but rather resorted to a brief description of the methods used in each of the studies.

### Conclusions

This scoping review provides a comprehensive assessment of the literature on the use of mobile and wearable AI in children with ASD, ADHD, and ID. Our scoping review found large heterogeneity of methods and findings in AI studies in child psychiatry. Overall, the largest gaps identified in this scoping review are the lack of randomized controlled trials, as most studies available were pilot trials. The definition of digital biomarkers used in these studies seems to be very wide. The studies included in the review had a small number of participants. Nevertheless, our scoping review identified several key strengths across the disorders considered in this study. First, wearable technologies comprising multiple sensors (eg, HR, sleep, and accelerometers) demonstrate promise in the diagnosis and prediction of aspects of disorders spanning child and adolescent psychiatry. Second, analytic solutions using data from wearables and expert annotations of child behavior can predict the onset of behavioral changes relevant to psychiatric disorders. Hence, given the growing ubiquity of wearables across the age span (children to parents, guardians, or teachers), our review strongly suggests the incorporation of wearables in child and adolescent psychiatry research. Such integration would be pivotal in facilitating remote monitoring and remote psychiatric services, which will likely help reduce disparities in mental health care access because of a shortage of child and adolescent psychiatrists. From a research perspective, the interaction of wearable biomarkers with conventional biomarkers (eg, genomics, metabolomics, neuroimaging, EEG, and environmental exposures) in the context of the diagnosis, treatment, and management of psychiatric disorders is yet to be pursued in large studies. Finally, the integration of wearables in child and adolescent psychiatry research should extend beyond controlled research settings to allow the extraction of the benefits of AI approaches. Naturalistic studies should look to collect annotations of children’s behavior as observed in their daily life at home, day care, or school and expand the involvement of relevant stakeholders in studies, wherein not only are parents annotating behaviors but also teachers, social workers, and counselors. Such a collection of annotated data from a real-world environment where children and adolescents develop will also provide opportunities to innovate with AI approaches such as reinforcement learning. Future directions should focus specifically on enrolling larger number of more diverse groups of patients. Future research should also focus on assessing which tools, mobile and wearable, are most efficient in collecting the most reliable data in various patient populations, as the primary outcome of interest.

## Appendix

Multimedia Appendix 1 PRISMA (Preferred Reporting Items for Systematic Reviews and Meta-Analyses) scoping review checklist.

Multimedia Appendix 2 Actual search strategies used in the study.

## Abbreviations

ADHD: attention-deficit/hyperactivity disorder
AI: artificial intelligence
ASD: autism spectrum disorder
ECG: electrocardiogram
EEG: electroencephalography
HR: heart rate
ID: internalizing disorder
NLP: natural language processing
PRISMA: Preferred Reporting Items for Systematic Reviews and Meta-Analyses
RSA: respiratory sinus arrhythmia
