# Supplementary material for: Use of Mobile and Wearable Artificial Intelligence in Child and Adolescent Psychiatry: Scoping Review
Source: J Med Internet Res. 2022 Mar 14;24(3):e33560. doi: 10.2196/33560 (PMC8961347; doi:10.2196/33560)
Supplement: Multimedia Appendix 2 [file jmir_v24i3e33560_app2.docx]

**Appendix 1. Actual Search Strategies**

**OVID**

Database(s): **Embase**1988 to 2021 Week 24**, Ovid MEDLINE(R) and Epub Ahead of Print, In-Process, In-Data-Review & Other Non-Indexed Citations**1996 to June 23, 2021**, EBM Reviews - Cochrane Central Register of Controlled Trials**May 2021**, EBM Reviews - Cochrane Database of Systematic Reviews**2005 to June 23, 2021
Search Strategy:

| **#** | **Searches** |
| --- | --- |
| 1 | Wearable Electronic Devices/ or exp wearable computer/ |
| 2 | wearable*.ti,ab,hw,kw. |
| 3 | ("smart watch*" or smartwatch* or "apple watch*" or "smart glasses" or smartglass* or "google glass*" or (smartphone* adj5 (app or intervention)) or "fitness tracker*" or "activity tracker*" or mHealth or "mobile app").ti. |
| 4 | *Smartphone/ |
| 5 | *Mobile Applications/ or *mobile application/ or (mHealth or "mobile app").ti,ab. |
| 6 | or/1-5 |
| 7 | Mental Disorders/ |
| 8 | Anxiety Disorders/ |
| 9 | mood disorders/ or mood disorder/ |
| 10 | depressive disorder/ or depression/ |
| 11 | depressive disorder, major/ or major depression/ |
| 12 | bipolar disorder/ |
| 13 | exp "attention deficit and disruptive behavior disorders"/ or attention deficit disorder/ |
| 14 | exp autism spectrum disorder/ |
| 15 | behavior disorder/ or conduct disorder/ or exp disruptive behavior/ or impulse control disorder/ or oppositional defiant disorder/ |
| 16 | Psychotic Disorders/ or psychosis/ |
| 17 | (psychiatry or psychiatric or psychotic or psychoses or anxiety or bipolar or depression or "learning disabilit*" or ADHD or "attention-deficit*" or "attention deficit*" or "oppositional defian*" or autism or autistic or asperger* or ((behavior* or conduct) adj3 (disorder* or dysfunction*))).ti. |
| 18 | exp Learning Disabilities/ or exp learning disorder/ |
| 19 | or/7-18 |
| 20 | 6 and 19 |
| 21 | (child* or pediatric* or paediatric* or young or youth or baby or babies or infant* or toddler* or teen* or adolescent* or adolescence).ti. |
| 22 | 20 and 21 |
| 23 | limit 20 to "all child (0 to 18 years)" [Limit not valid in Embase,CCTR,CDSR; records were retained] |
| 24 | Psychology, Child/ or (child* adj2 psych*).ti,ab. |
| 25 | child behavior disorders/ or (child* adj2 (conduct or behavior*) adj2 (disorder* or dysfunction)).ti,ab. |
| 26 | 24 or 25 |
| 27 | 6 and 26 |
| 28 | 22 or 23 or 27 |
| 29 | (exp animals/ or exp nonhuman/) not exp humans/ |
| 30 | ((alpaca or alpacas or amphibian or amphibians or animal or animals or antelope or armadillo or armadillos or avian or baboon or baboons or beagle or beagles or bee or bees or bird or birds or bison or bovine or buffalo or buffaloes or buffalos or "c elegans" or "Caenorhabditis elegans" or camel or camels or canine or canines or carp or cats or cattle or chick or chicken or chickens or chicks or chimp or chimpanze or chimpanzees or chimps or cow or cows or "D melanogaster" or "dairy calf" or "dairy calves" or deer or dog or dogs or donkey or donkeys or drosophila or "Drosophila melanogaster" or duck or duckling or ducklings or ducks or equid or equids or equine or equines or feline or felines or ferret or ferrets or finch or finches or fish or flatworm or flatworms or fox or foxes or frog or frogs or "fruit flies" or "fruit fly" or "G mellonella" or "Galleria mellonella" or geese or gerbil or gerbils or goat or goats or goose or gorilla or gorillas or hamster or hamsters or hare or hares or heifer or heifers or horse or horses or insect or insects or jellyfish or kangaroo or kangaroos or kitten or kittens or lagomorph or lagomorphs or lamb or lambs or llama or llamas or macaque or macaques or macaw or macaws or marmoset or marmosets or mice or minipig or minipigs or mink or minks or monkey or monkeys or mouse or mule or mules or nematode or nematodes or octopus or octopuses or orangutan or "orang-utan" or orangutans or "orang-utans" or oxen or parrot or parrots or pig or pigeon or pigeons or piglet or piglets or pigs or porcine or primate or primates or quail or rabbit or rabbits or rat or rats or reptile or reptiles or rodent or rodents or ruminant or ruminants or salmon or sheep or shrimp or slug or slugs or swine or tamarin or tamarins or toad or toads or trout or urchin or urchins or vole or voles or waxworm or waxworms or worm or worms or xenopus or "zebra fish" or zebrafish) not (human or humans or patient or patients)).ti,ab,hw,kw. |
| 31 | (rat or rats or mice or mouse or murine or pig or pigs or porcine or swine or dog or dogs).ti. |
| 32 | or/29-31 |
| 33 | 28 not 32 |
| 34 | limit 33 to (english language and yr="2011 -Current") [Limit not valid in CDSR; records were retained] |
| 35 | remove duplicates from 34 |

**SCOPUS**

| 1 | - TITLE ("smart watch*" or smartwatch* or "apple watch*" or "smart glasses" or smartglass* or "google glass*" or (smartphone* W/5 (app or intervention)) or "fitness tracker*" or "activity tracker*" or mHealth or "mobile app") |
| --- | --- |
| 2 | TITLE (psychiatry or psychiatric or psychotic or psychoses or anxiety or bipolar or depression or "learning disabilit*" or ADHD or "attention-deficit*" or "attention deficit*" or "oppositional defian*" or autism or autistic or asperger* or ((behavior* or conduct) W/3 (disorder* or dysfunction*))) |
| 3 | TITLE-ABS-KEY (child* or pediatric* or paediatric* or young or youth or baby or babies or infant* or toddler* or teen* or adolescent* or adolescence) |
| 4 | 1 and 2 and 3 |
| 5 | INDEX(embase) OR INDEX(medline) OR PMID(0* OR 1* OR 2* OR 3* OR 4* OR 5* OR 6* OR 7* OR 8* OR 9*) |
| 6 | 4 not 5 |

**Web of Science**

| 1 | TS=("smart watch*" or smartwatch* or "apple watch*" or "smart glasses" or smartglass* or "google glass*" or (smartphone* NEAR/5 (app or intervention)) or "fitness tracker*" or "activity tracker*" or mHealth or "mobile app") |
| --- | --- |
| 2 | TS=(psychiatry or psychiatric or psychotic or psychoses or anxiety or bipolar or depression or "learning disabilit*" or ADHD or "attention-deficit*" or "attention deficit*" or "oppositional defian*" or autism or autistic or asperger* or ((behavior* or conduct) NEAR/3 (disorder* or dysfunction*))) |
| 3 | TS=(child* or pediatric* or paediatric* or young or youth or baby or babies or infant* or toddler* or teen* or adolescent* or adolescence) |
| 4 | 1 and 2 and 3 |
| 5 | PMID=(0* or 1* or 2* or 3* or 4* or 5* or 6* or 7* or 8* or 9*) |
| 6 | 4 not 5 |
